# Clinical Data, Comorbidities, and Mortality of COVID-19 in the State of Guanajuato, Mexico until May 20, 2020

**DOI:** 10.5195/cajgh.2020.527

**Published:** 2020-03-31

**Authors:** Nicolás Padilla-Raygoza, Efraín Navarro-Olivos, María de Jesús Gallardo-Luna, Francisco J. Magos-Vázquez, Daniel Alberto Díaz-Martínez, Cuauhtémoc Sandoval-Salazar, Luis Antonio Díaz-Becerril

**Affiliations:** 1 School of Medicine, University of Celaya, Celaya Mexico; 2 Institute of Public Health from Guanajuato State, Guanajuato, Mexico; 3 Department of Nursing and Obstetrics, Division of Health Sciences and Engineering, University of Guanajuato, Celaya, Mexico

**Keywords:** SARS-CoV-2, COVID-19, Population, Deaths, Clinical data, Comorbidities

## Abstract

**Introduction:**

In December 2019, cases of pneumonia of unknown cause arose in Wuhan, China. The causative agent was subsequently identified as 2019-nCoV and later called SARS-CoV-2. In Mexico, since January 2020 when the first cases were reported, the spread of the infection has occurred throughout the country. The state of Guanajuato, which is located in the center of the country, has taken isolation measures and closed public places in March 2020. The objective of this study was to analyze the evolution, symptoms, co-morbidities and deaths due to confirmed cases of COVID-19.

**Methods:**

An ecological study was designed from the database of confirmed cases of COVID-19 in the state of Guanajuato. Odds ratios and 95% confidence intervals were calculated for symptoms and co-morbidities in deaths of confirmed cases. Logistic regression models were generated adjusting for age group and gender.

**Results:**

Among the 838 confirmed cases in the state, cases with dyspnea and cyanosis showed more significant effect on death. Age group and gender had little involvement as confounders. For practically all comorbidities (including diabetes, hypertension, cardiovascular disease, chronic kidney disease, and immunosuppression), there was a significant effect (odds ratio greater than 2) on mortality from COVID-19. Age group showed a confounding effect on comorbidities and death, but not gender.

**Conclusions:**

The confirmed cases had more than twice the possibility of having comorbidities, compared with those who did not die.

At the end of 2019, the World Health Organization (WHO) office in Wuhan, Hubei Province, China, received the report of a case of pneumonia of unknown cause^1^ that was related to other cases of pneumonia in people who worked or lived near the local Hunan seafood market^2^-^5^. The infection spread throughout the city and to other countries and was declared an international public health emergency by the WHO^6^. The causative agent was determined to be a new coronavirus, called 2019-nCoV and later SARS-CoV-2 by WHO^7^. It was reported that the cause of COVID-19 shares 79.5% of the SARS-CoV sequence and uses the same cell entry receptor, angiotensin-converting enzyme-2, as SARS-CoV^8^. Zhu et al.^9^ reported the cytopathic effects and morphology of the virus and that it is a member of a family of coronaviruses that infect humans. This virus grew more in human airway epithelial cells than tissue culture cells, suggesting the potential for increased infectivity.

COVID-19 patients who present with a comorbid condition may have an increased risk of deterioration and should therefore be admitted to a designated unit for close monitoring in accordance with the WHO guidelines for screening and triage^10^. In a series of 41 patients infected with SARS-CoV-2, 32% had some underlying pathology, 20% had diabetes, 15% had hypertension, 15% had cardiovascular disease, and 2% had chronic obstructive pulmonary disease (COPD)^11^.

In Mexico, the first detected case started with symptoms on January 8, 2020, and the first two deaths were reported on March 18, 2020^12^. In the state of Guanajuato, before the arrival of the pandemic, the local authorities began measures of social isolation. The closure of educational institutions at all levels began on March 20, 2020, and later, there was the closure of restaurants, gyms, and public parks. Meetings with more than 10 people were also cancelled.

The state of Guanajuato is located in the center of the Mexican Republic (Longitude #102°5'49.2” W #99° 40'16.68" W, Latitude 19° 54'46.08" N 21° 50'21.84" N^13^). As of the 2010 Mexican census, Guanajuato had 5,486,372 inhabitants, accounting for 4.88% of the national population^14^. The state is a relay center for transportation to the four cardinal points of Mexico. In the state of Guanajuato, the first confirmed case of COVID-19 was reported with the onset of symptoms on March 10, 2020, and the first two deaths were reported on April 5, 2020^15^. The number of confirmed cases of COVID-19 in Guanajuato State remained low through April, but given the significant dates in May for the Mexican population, people began to break social isolation, and the number of confirmed cases increased markedly.

The aim of this study was to analyze the effect of clinical data and comorbidities on deaths from COVID-19 in Guanajuato State, Mexico. This is important because few studies are published from Mexico and even less so from Guanajuato State.

## Methods

An analytical ecological study was designed with the data reported in the database^15^ of the Secretary of Health of the State of Guanajuato, with confirmed cases and deaths from COVID-19 until May 20, 2020. A suspected case is one that manifests fever, cough, dyspnea, and has had a trip abroad to a country with a high frequency of COVID-19 cases or have had contact with a confirmed case. A confirmed case is one that, in addition to the previous criteria, also tested positive for the virus using reverse transcriptase-polymerase chain reaction (RT-PCR).

Sociodemographic variables in the database were age and gender. Among the study variables, the date of onset of symptoms was collected, as well as the clinical data recorded: fever, cough, dyspnea, odynophagia, diarrhea, vomiting, headache, chest pain, cyanosis, abdominal pain, myalgia, arthralgia, and rhinorrhea. All were measured as absent or present. Other variables were comorbidities in the confirmed patient: diabetes, chronic obstructive pulmonary disease (COPD), asthma, immunosuppression, hypertension, cardiovascular disease, chronic kidney disease, obesity, and smoking. The result variable was death and its date. All included registries had a positive RT-PCR test for SARS-CoV-2.

For statistical analysis, descriptive statistics were used to show the variables. Odds ratios (OR) and corresponding 95% confidence intervals (95% CI) were used for clinical data, comorbidities, and COVID-19 death. Logistic regression models were generated between comorbidities and death by COVID-19, using age categories and gender as potential confounding variables. Statistical analyses were performed using STATA 13.0 ® (Stata Corp., College Station, TX, USA).

## Results

The sample consisted of 838 confirmed cases distributed throughout the state, with the municipality of León predominating. The distribution by gender was 378 (45.11%) women and 460 (54.89%) men. Ages ranged from 0 to 93 years, with an average of 45.03± 17.76 years.

**Figure 1.**
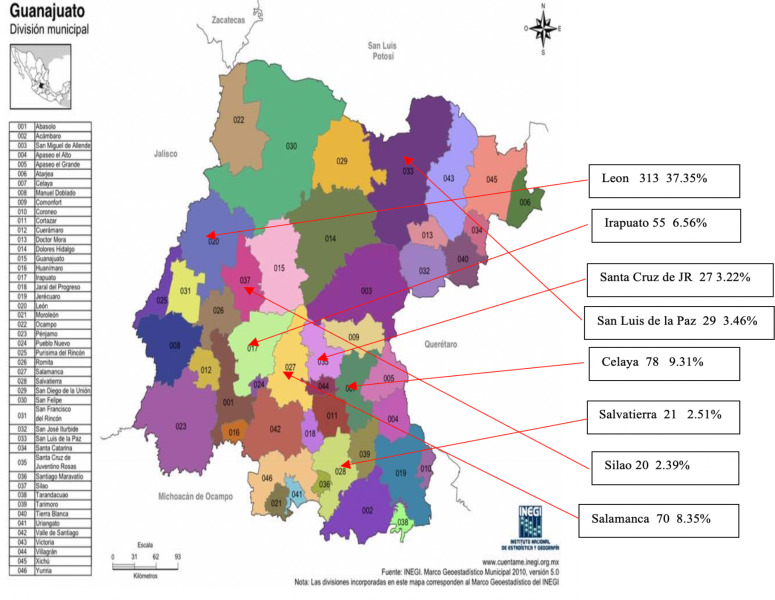
Map of Guanajuato State, with municipalities with 20 or more confirmed cases of COVID-19

Source: INEGI^14^ and SINAVE/DGE^15^

Figure 1 shows the municipalities of the state of Guanajuato with a report of at least 20 cases; the rest of the municipalities had at least one case reported.

**Figure 2.**
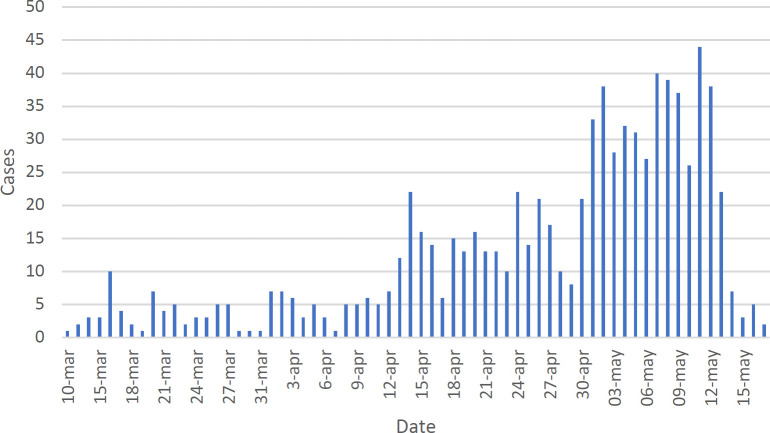
Distribution of confirmed cases of COVID-19 by day in Guanajuato State, Mexico (n-848)

Source: SINAVE/DGE^15^

**Figure 3.**
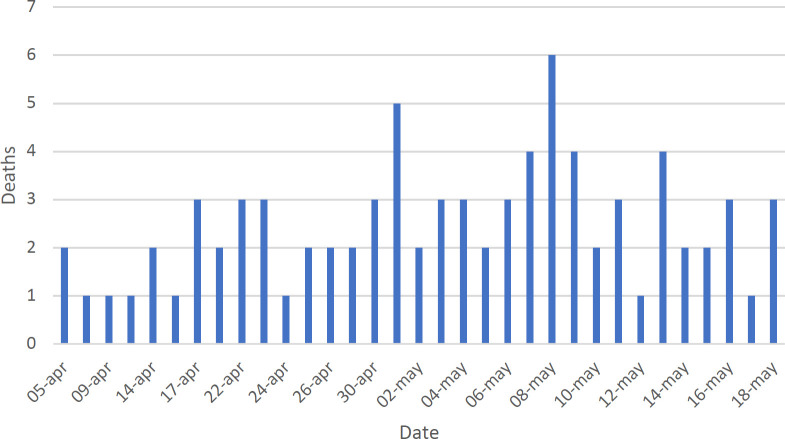
Distribution of deaths for COVID-19 by day in Guanajuato State, Mexico (n=80)

Source: SINAVE/DGE^15^

Figure 2 shows the distribution of confirmed cases of COVID-19 per day. The first detected case in Guanajuato State started with symptoms on March 10, 2020 and the curve remained low, possibly due to the measures of social isolation and closure of public places that began to be applied in the state as of March 17. Throughout April, however, the curve for cases increased. The few confirmed cases in mid-May may be an artifact due to delayed delivery of RT-PCR test results for SARS-CoV-2. Figure 3 shows the distribution of deaths due to COVID-19 per day. The specific mortality rate (SMR=9.55%) for Guanajuato until May 20, 2020 is 80 deaths among 838 cases.

Table 1 shows the distribution by age and gender of confirmed cases for COVID-19 by death (n=838). Men accounted for 62.50% of the deaths by COVID-19, though among the non-deceased, accounted for a similar but lower percentage of 54.09%. The OR for gender indicates that being a woman was a protective factor for dying by decreasing the risk by 29%, an effect that is nullified when reviewing the 95% CI. For age group, those aged 60 years or older predominated among the deceased (57.50%).

**Table 1.** Distribution of gender and age group by death for COVID-19 in Guanajuato, Mexico

Confirmed cases					
Variable	Deaths (n=80)		Non-deaths (n=758)		OR (95% CI)
	n	%	n	%	
**Gender**					0.71 (0.44 to 1.14)
Female	30	37.50	348	45.91	
Male	50	62.50	410	54.09	
**Age group (years)**					3.59 (2.66 to 4.85)
0–5	0	0	14	1.85	
6–11	0	0	10	1.32	
12–19	0	0	24	3.17	
20–49	12	15.00	444	58.58	
50–59	22	27.50	145	19.13	
60 or higher	46	57.50	121	15.96	

Presence of dyspnea and cyanosis had the greatest increased risk of death from COVID-19 compared to those who were not deceased. The deceased had more than ten times the odds of having presented dyspnea or cyanosis than those not deceased. Fever and chest pain only had an effect on death four times greater.

Diarrhea, cough, vomiting, headache, myalgia, arthralgia, and rhinorrhea had no statistically significant effect on mortality. Sore throat had a protective effect against death. Age group had a confounding effect for dyspnea, chest pain, and cyanosis, but maintained the strong effect of these clinical data on death (Table 2).

**Table 2.** Distribution of clinical data by death from COVID-19 in Guanajuato, Mexico

Clinical data	Confirmed cases (n=838)				Logistic regression OR (95% CI)		
	Deaths		Non-deaths		Unadjusted	Age-adjusted	Gender-adjusted
	n	%	n	%			
**Fever** ^*^					4.17 (1.78 to 9.72)	4.19 (1.75 to 10.05)	4.02 (1.72 to 9.41)
Yes	74	92.50	566	74.77			
No	6	7.50	191	25.23			
**Cough**					1.21 (0.57 to 2.60)	1.18 (0.53 to 2.65)	1.23 (0.57 to 2.65)
Yes	72	90.00	668	88.13			
No	8	10.00	90	11.87			
**Sore throat**					0.37 (0.23 to 0.60)	0.48 (0.29 to 0.79)	0.38 (0.24 to 0.61)
Yes	30	37.50	466	61.48			
No	50	62.50	292	38.52			
**Dyspnea**					18.80 (10.28 to 34.37)	11.01 (5.88 to 20.60)	18.73 (10.23 to 34.36)
Yes	66	82.50	152	20.05			
No	14	17.50	606	79.95			
**Diarrhea**					2.15 (1.23 to 3.75)	1.84 (1.01 to 3.37)	2.08 (1.19 to 3.64)
Yes	19	23.75	96	12.66			
No	61	76.25	662	87.34			
**Voinitiug**					1.23 (0.51 to 2.97)	1.54 (0.60 to 3.98)	1.22 (0.50 to 2.95)
Yes	6	7.50	47	6.20			
No	74	92.50	711	93.80			
**Headache**					0.71 (0.39 to 1.29)	0.75 (0.39 to 1.44)	0.72 (0.39 to 1.30)
Yes	65	81.25	651	85.88			
No	15	18.75	107	14.12			
**Chest paiu** ^*^					3.82 (2.38 to 6.13)	2.61 (1.58 to 4.31)	3.96 (2.46 to 6.38)
Yes	40	50.00	157	20.74			
No	40	50.00	600	79.26			
**Abdominal pain**					1.31 (0.63 to 2.73)	1.04 (0.48 to 2.28)	1.32 (0.63 to 2.76)
Yes	9	11.25	67	8.84			
No	71	88.75	691	91.16			
**Myalgias** ^*^					1.12 (0.68 to 1.87)	1.29 (0.75 to 2.22)	1.09 (0.66 to 1.82)
Yes	57	71.25	521	68.82			
No	23	28.75	236	31.18			
**Arthralgias**					1.41 (0.87 to 2.29)	1.23 (0.74 to 2.07)	1.40 (0.86 to 2.27)
Yes	53	66.25	441	58.18			
No	27	33.75	317	41.82			
**Rhinorrhea**					0.80 (0.50 to 1.30)	1.00 (0.60 to 1.68)	0.82 (0.51 to 1.33)
Yes	28	35.00	304	40.11			
No	52	65.00	454	59.89			
**Cyanosis** ^**^					13.25 (6.19 to 28.37)	7.66 (3.34 to 17.53)	13.81 (6.40 to 29.78)
Yes	16	20.00	14	1.85			
No	64	80.00	742	98.15			

* One case removed for missing information

** Two cases removed for missing information

Source: SINAVE/DGE^15^

Asthma showed a non-significant protective effect. Diabetes, hypertension, COPD, cardiovascular disease, and chronic kidney disease show a strong effect on mortality from COVID-19 with ORs greater than 3. For each of these comorbidities, age group acted as a confounder, decreasing the ORs, but they remained significant. For obesity and smoking, an OR effect was found higher than 2, though age group also acted as a confounder. Gender in no one comorbidities acted as a confounder (Table 3).

**Table 3.** Distribution among comorbidities by deaths from COVID-19 in Guanajuato, Mexico

Comorbidity	Confirmed cases (n=838)				Logistic regression OR (95% CI)		
	Deaths		Non-deaths		Unadjusted	Age-adjusted	Gender-adjusted
	n	%	n	%			
**Diabetes**					3.10 (1.84 to 5.22)	1.29 (0.73 to 2.28)	3.17 (1.88 to 5.34)
Yes	25	31.25	97	12.80			
No	66	68.75	661	87.20			
**Hypertension**					5.98 (3.68 to 9.71)	2.57 (1.50 to 4.40)	6.01 (3.70 to 9.78)
Yes	39	48.75	104	13.72			
No	41	51.25	654	86.28			
**COPD**					4.93 (2.15 to 11.30)	1.54 (0.64 to 3.69)	5.06 (2.20 to 11.66)
Yes	9	11.25	19	2.51			
No	71	88.75	739	97.49			
**Asthma**					0.86 (0.11 to 6.75)	0.72 (0.08 to 6.33)	0.85 (0.1 Ito 6.68)
Yes	1	1.25	11	1.45			
No	79	98.75	747	98.55			
**Cardiovascular disease** ^*^					4.01 (1.51 to 10.65)	1.57 (0.56 to 4.45)	4.07 (1.53 to 10.85
Yes	6	7.50	15	1.98			
No	74	92.50	742	98.02			
**Immunosuppression**					N/A	N/A	N/A
Yes	0	0.00	9	1.19			
No	80	100.00	749	98.81			
**C hronic kidney disease**					4.18 (1.06 to 16.49)	3.19 (0.71 to 14.28	4.13 (1.04 to 16.37)
Yes	3	3.75	7	0.92			
No	77	96.25	751	99.08			
**Obesity**					2.37 (1.41 to 3.98)	2.19 (1.25 to 3.84)	2.44 (1.45 to 4.11)
Yes	24	30.00	116	15.30			
No	56	70.00	642	84.70			
**Smoking**					2.43 (1.27 to 4.67)	1.76 (0.86 to 3.59)	2.37 (1.23 to 4.56)
Yes	13	6.25	56	7.39			
No	67	83.75	702	92.61			

* One case removed for missing information

Source: SINAVE/DGE^15^

## Discussion

The sample of 838 infected with SARS-CoV-2 who developed COVID-19 registered in the state of Guanajuato shows a very slow spread during the month of March, but in April and May, the number of cases increased markedly (Figure 2). This slow spread is possibly due to the initial measures taken by the government of the state for social isolation and closure of public places, as well as the avoidance of massive events. As the quarantine and social isolation continued, the population may have gone out to cover basic needs and obtain food supplies, and in places of supply possibly being infected, infection may have spread. This could explain the increase in cases in the months of April and May despite social isolation. It should not be forgotten that the RT-PCR test only applies to symptomatic patients, so asymptomatic carriers are not detected and may avoid their isolation.

It was reported that after social distancing, 20% of new cases and many hospitalizations could be avoided, but upon completion of isolation, new cases would rebound^16^ and, reinforced by Li et al.^17^, that the imposition of social controls impacts the number of new cases.

The SMR of 9.55% in the state of Guanajuato is slightly lower than that of Mexico, which was 10.59% as of May 15, 2020^18^, but is higher than the global SMR of 6.34% reported by the WHO^19^.

The most reported symptoms among the Guanajuato cases were fever, cough, dyspnea, and sore throat, but the symptoms that showed the greatest effect in terms of mortality were cyanosis, dyspnea, and chest pain (Table 2). In a series of 926 symptomatic SARSCoV-2 infected cases, there was fever reported in 42.2% of cases, cough in 67.2%, dyspnea in 15.0%, arthralgia in 14.4%, headache in 13.4%, sore throat in 14.0%, diarrhea in 3.3%, and vomiting in 4.6%^20^. The figures reported in patients from Guanajuato, Mexico differ from these reported by Guan et al.^20^, but the predominant symptoms were still fever, cough, and dyspnea. Liu et al.^21^, in a series of 44 symptomatic patients with COVID-19, reported fever in 97.7%, cough in 56.8%, dyspnea in 9.1%, and arthralgia in 52.3%. In a series of nine pregnant women, fever was reported in 77.8%, cough in 44.4%, dyspnea in 11.1%, arthralgia in 33.3%, headache in 33.3%, and sore throat in 33.3%^22^.

The comorbidities with the greatest effect on mortality in patients from the state of Guanajuato are similar (Table 3) to those described in all of Mexico^18^. In a series of 41 patients infected with SASR-CoV-2 in Wuhan, China, 32% had some underlying pathology, 20% had diabetes, 15% had hypertension, 15% had cardiovascular disease, and 2% had COPD^23^.

The spread of SARS-CoV-2 infection has been constantly increasing since January 2020. In the state of Guanajuato, since mid-March, the government authorities decided to close educational institutions, mass events, and public places, which resulted in the curve for new confirmed cases remaining low; with community transmission, cases increased dramatically during April and May. The clinical data of the confirmed cases in the state of Guanajuato are similar to those already reported, with cough, fever, and dyspnea as the main symptoms. Mortality in the presence of comorbidities such as diabetes, hypertension, COPD, and cardiovascular disease in the state of Guanajuato are similar to what is reported throughout Mexico. It is important to continue the follow-up of the epidemiology of SARS-CoV-2 in Guanajuato State because it is possible that the number of confirmed cases may rise with the breaking of quarantine.

## References

[1] World Health Organization. Rolling updates on coronavirus disease (COVID-19). World Health Organization. https://www.who.int/emergencies/diseases/novel-coronavirus-2019/events-as-they-happen. Updated 7 May 2020. Accessed: June 21, 2020.

[2] Lu H, Stratton CW, Tang YW. Outbreak of pneumonia of unknown etiology in Wuhan, China: the mystery and the miracle. *J Med Virol*. 2020; 92(4):401–402. Doi: 10.1002/jmv.25678 31950516 PMC7166628

[3] Hui DS, Azhar EI, Madani TA, Ntoumi F, Kock R, Dar O, et al. The continuing 2019-nCoV epidemic threat of novel coronaviruses to global health – the latest 2019 novel coronavirus outbreak in Wuhan, China. *Int J Infect Dis*. 2020; 91: 264–66 Doi: 10.1016/j.ijid.2020.01.009 31953166 PMC7128332

[4] Huang C, Wang Y, Li X, Ren L, Zhao J, Hu Y, et al. Clinical features of patients infected with 2019 novel coronavirus in Wuhan, China. *The Lancet*. 2020; 395(10223):497–506 Doi: 10.1016/S0140-6736(20)30183-5 PMC7159299 31986264

[5] Chen N, Zhou M, Dong X, Qu J, Gong F, Han Y, et al. Epidemiological and clinical characteristics of 99 cases of 2019 novel coronavirus pneumonia in Wuhan, China: a descriptive study. *The Lancet*. 2020; 395(10223): 507–513 10.1016/S0140-6736(20)30211-7 PMC7135076 32007143

[6] Yoo JH, Hong ST. The outbreak cases with the novel coronavirus suggest upgraded quarantine and isolation in Korea. *J Korean Med Sci*. 2020;35(5): e62 Doi: 10.3346/jkms.2020.35.e62 32030926 PMC7008072

[7] Carlos WG, De la Cruz C, Cao B, Pasnick S, Jamil S. Novel Wuhan (2019-CoV) coronavirus. *Am J Respi Crit Care Med*. 2020;201(4): 7–8 Doi: 10.1164/rccm.2014P7 32004066

[8] Zhou P, Yang XL, Wang XG, Hu B, Zhang L, Zhang W, et al. A pneumonia outbreak associated with a new coronavirus of probable bat origin. *Nature*. 2020; 579: 270–273. 10.1038/s41586-020-2012-7 32015507 PMC7095418

[9] Zhu N, Zhang D, Wang W, Li X. Yang B, Song J, et al. A novel coronavirus from patients with pneumonia in China, 2019. *N Engl J Med*. 2020; 382: 727:737. Doi: 10.1056/NEJMoa2001017 31978945 PMC7092803

[10] World Health Organization. Clinical management of severe acute respiratory infection (SARI) when COVID-19 disease is suspected. Interim guidance. World Health Organization. 2020. https://www.who.int/publications-detail/clinical-management-ofsevere-acute-respiratory-infection-when-novel-coronavirus-(ncov)-infection-is-suspected.. Accessed June 21, 2020.

[11] Huang C, Wuang Y, Li X, Ren L, Zhao J, Hu Y, et al. Clinical features of patients infected with 2019 novel coronavirus in Wuhan, China. *The Lancet*. 2020; 395(10223): 497–506 Doi: 10.1016/S0140-6736(20)30183-5 PMC7159299 31986264

[12] Secretaría de Salud. Datos-Abiertos Bases históricas. 6 mayo 2020. Available in: https://www.gob.mx/salud/documentos/datos-abiertos-bases-historicas-direccion-general-de-epidemiologia Accessed May 20, 2020

[13] INEGI. Mexico en Cifras. Guanajuato. INEGI. https://www.inegi.org.mx/app/areasgeograficas/?ag=11, Accessed June 21, 2020

[14] INEGI-Población. INEGI. https://www.inegi.org.mx/temas/estructura/.. Accessed: June 21, 2020

[15] Departamento de Epidemiología de la Dirección de Servicios de Salud. Sistema Nacional de Vigilancia Epidemiológica, Dirección General de Epidemiología, Secretaría de Salud. Available in: http://www.sinave.gob.mx/.2020.. Accessed May 21, 2020.

[16] Matrajt L, Leung T. Evaluating the effectiveness of social distancing interventions to delay or flatten the epidemic curve of Coronavirus disease. *Emerg. Infect. Dis*. 2020; 26(8). Doi: 10.3201/eid2608.201093 PMC7392458 32343222

[17] Li L, Yang Z, Dang Z, Meng C, Huang J, Meng H, et al. Propagation analysis and prediction of the COVID-19. *Infectious Disease Modelling*. 2020; 3: 282–292. Doi: 10.1016/j.idm.2020.03.002 PMC7118312 32292868

[18] Padilla-Raygoza N, Sandoval-Salazar C, Díaz-Becerril LA, Beltran-Campos V, Díaz-Martínez DA, Navarro-Olivos E, et al. Update of the Evolution of SARS-CoV-2 infection, COVID-19, and mortality in Mexico until May 15, 2020: An Ecological Study. *International Journal of Tropical Disease & Health*, 2020; 41(5): 36–45. Doi: 10.9734/IJTDH/2020/v41i/530277.

[19] World Health Organization. Coronavirus disease 2019 (COVID-19). Situation Report – 85, April 14, 2020. Available in: https://www.who.int/docs/default-source/coronaviruse/situation-reports/20200414-sitrep-85-covid-19.pdf?sfvrsn=7b8629bb_4 Accessed: June 21, 2020.

[20] Guan WJ, Ni ZY, Hu Y, Laing WH, Ou CQ, He JX, et al. Clinical Characteristics of Coronavirus Disease 2019 in China. *N Engl J Med*. 2020; 382:1708–1720. Doi: 10.1056/NEJMoa2002032 32109013 PMC7092819

[21] Liu J, Liu Y, Xiang P, Pu L, Xiong H, Li C, et al. Neutrophil-to-lymphocyte ratio predicts severe illness patients with 2019 novel coronavirus in the early stage. *medRxiv*. 2020. Doi: 10.1101/2020.02.10.20021584 PMC7237880 32434518

[22] Chen H, Gua J, Wang C, Lua F, Yu X, Zhang W, et al. Clinical characteristics and intrauterine vertical transmission potential of COVID-19 infection in nine pregnant women: a retrospective review of medical records. *The Lancet*. 2020; 395 (10226): 809–815. Doi: 10.1016/S0140-6736(20)30360-3 PMC7159281 32151335

[23] Huang C, Wuang Y, Li X, Ren L, Zhao J, Hu Y, et al. Clinical features of patients infected with 2019 novel coronavirus in Wuhan, China. *The Lancet*. 2020;395(10223): 497–506 Doi: 10.1016/S0140-6736(20)30183-5 PMC7159299 31986264

